# Synthesis of Novel Perfluoroalkylglucosides on Zeolite and Non-Zeolite Catalysts

**DOI:** 10.3390/molecules20046140

**Published:** 2015-04-08

**Authors:** Janusz Nowicki, Łukasz Mokrzycki, Bogdan Sulikowski

**Affiliations:** 1 Institute of Heavy Organic Synthesis “Blachownia”, Energetyków St. 9, 47-225 Kędzierzyn-Koźle, Poland; 2 Jerzy Haber Institute of Catalysis and Surface Chemistry, Polish Academy of Sciences, Niezapominajek St. 8, 30-239 Kraków, Poland; E-Mail: ncmokrzy@cyf-kr.edu.pl

**Keywords:** perfluoroalkylglucosides, Fischer reaction, zeolites, ion-exchange resin, montmorillonite

## Abstract

Perfluoroalkylglucosides comprise a very important class of fluorine-containing surfactants. These compounds can be synthesized by using the Fisher reaction, starting directly from glucose and the required perfluoroalcohols. We wish to report on the use of zeolite catalysts of different structure and composition for the synthesis of perfluoroalkylglucosides when using glucose and 1-octafluoropentanol as substrates. Zeolites of different pore architecture have been chosen (ZSM-5, ZSM-12, MCM-22 and Beta). Zeolites were characterized by XRD, nitrogen sorption, scanning electron microscopy (SEM) and solid-state ^27^Al MAS NMR spectroscopy. The activity of the zeolite catalysts in the glycosidation reaction was studied in a batch reactor at 100 °C below atmospheric pressure. The performance of zeolites was compared to other catalysts, an ion-exchange resin (Purolite) and a montmorillonite-type layered aluminosilicate. The catalytic performance of zeolite Beta was the highest among the zeolites studied and the results were comparable to those obtained over Purolite and montmorillonite type catalysts.

## 1. Introduction

Different carbohydrates, exemplified by glucose, can be alkylated by long-chain alcohols over acid catalysts to yield non-ionic surfactants. Sulfonic acids are used as catalysts on an industrial scale, but the glycosidation process continues to be studied using different classes of catalysts. Thus, dealuminated Y type zeolites were screened in glycosidation reactions [1]. Extensive studies on the use of zeolites in the synthesis of alkyl glucosides were conducted by Corma *et al.* [2,3,4,5]. It was demonstrated that the highest activity in the synthesis of both C_4_ and long-chain alkyl glucosides was exhibited by H-Beta zeolite. Glycosidation of disaccharides with short- and long-chain alcohols over MCM-41 mesoporous molecular sieves was also studied [6]. 

On the other hand, perfluoroalkylglucosides comprise a very specific and important class of fluorosurfactants due to their potential applications [7]. A detailed account of these surface active agents was given by Reiss and Greiner [8,9,10]. Their particular importance stems from their application as emulsion stabilizers in biomedicine. Emulsions of fluorohydrocarbons are excellent oxygen carriers, and they are also used as contrasting media in diagnostics and various systems for other medical applications. Stabilization of such emulsions can be accomplished by fluorinated surfactants, especially those based on various carbohydrates. However, before application of such compounds certain conditions must be met: first, a structure should be well-defined, second, their biocompatibility should be assessed taking into account the required purity. The criteria mentioned above have determined the majority of synthesis methods, chosen in such a way as to yield particular compounds amenable for non-problematic purification. 

One of synthesis paths yielding alkylglucosides with well-defined structures and free from contamination with higher oligomers is known as the Koenigs-Knorr method. In this method bromides of acetylated carbohydrates and a suitable alcohol are used as reagents (after deacetylation, a glucoside is obtained). However, when using fluorinated alcohols, an abnormal synthesis route ocurrs, and, instead of a glucoside, an orthoester is formed; detailed accounts of this phenomenon were given by Riess *et al.* [8]. As concluded by Riess, the very low nucleophilicity of perfluoroalcohols makes it difficult to substitute the C1 hydroxy group in the glucose molecule and form perfluoroalkyl glucosides using the Koenigs-Knorr route. High yields of O-fluoroalkylglucosides (>90%) were obtained when using this method. Its modification is based on formation of 1-O-alkylglucoside, which reacts further with a fluoroalkyl iodide to yield a iodoperfluoroglucoside. The latter compound can be conveniently transformed into a perfluoroalkylglucoside by zinc reduction. 

The Mitsunobu reaction has gained wide acceptance as a versatile method in organic synthesis—it transforms hydroxyl groups into another groups which can be in turn displaced by nucleophiles [11]. Thus, *O*-fluoroalkylglucosides can be readily prepared according to this route. Synthesis of perfluoroalkylglucosides was carried out for the first time by Falck [12].

**Scheme 1:**
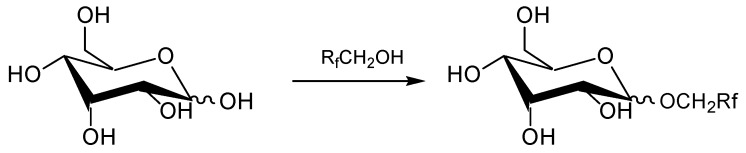
Synthesis of perfluoroalkylglucosides from glucose and alcohols by using the Fischer reaction.

The methods described above have, however, limited application for laboratory use only. In this contribution we propose a useful method for the synthesis of perfluoroalkylglucosides by applying a Fischer reaction, that is, starting directly from glucose and the corresponding perfluoroalcohol (*cf.* Scheme 1). The results of glycosidation by the Fischer reaction over a variety of catalysts were described by Straathof *et al.*, in particular, the acidic ion-exchange resins (Amberlyst 131, Purolite C122) proved to be especially good for this purpose [13]. 

The objective of the present work was to apply zeolite catalysts in the reaction visualized in Scheme 1. Four zeolites differing in structure, composition and pores architecture have been chosen: ZSM-5, ZSM-12, MCM-22 and Beta. Catalytic tests were performed in the liquid phase under the conditions relevant for practical implementation.

## 2. Results and Discussion 

### 2.1. Catalyst Characterization

#### 2.1.1. ZSM-12

The framework density of silicon and aluminium atoms is 19.4 T atoms/1000 Å^3^ unit cell and there are 56 T-atoms in the unit cell. The channel system of the ZSM-12 zeolite runs along the *010* direction, belonging to a 12-ring pore system with openings of 5.7 × 6.1 Å. 

**Figure 1:**
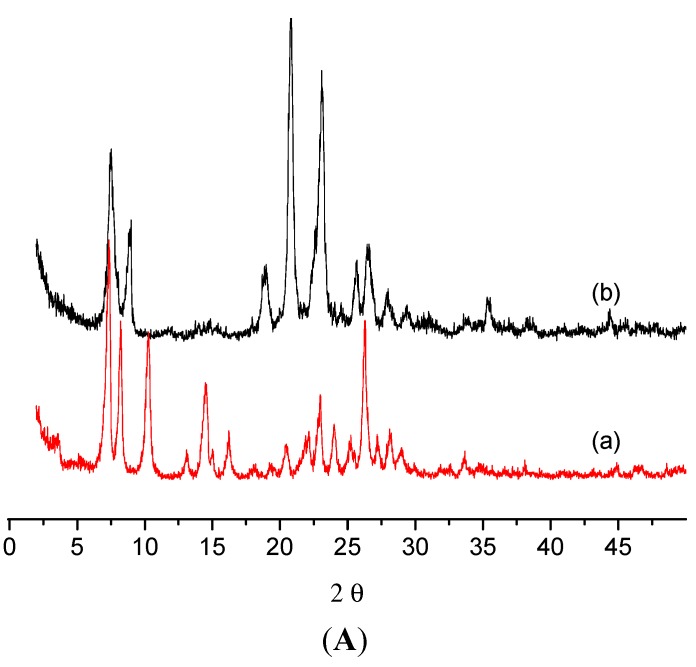

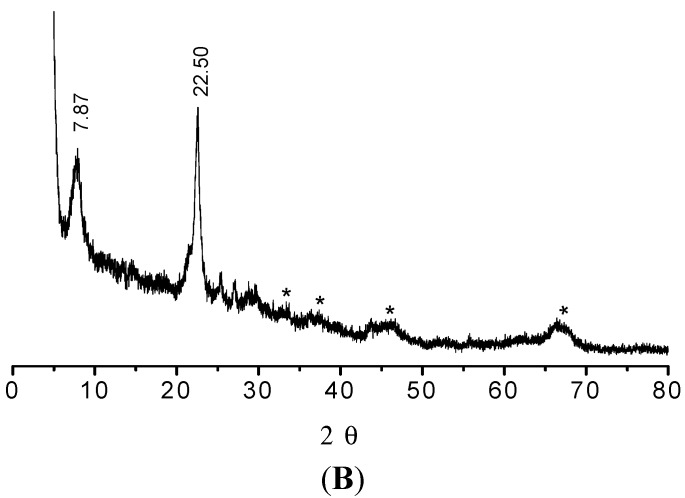
(**A**) X-ray diffraction patterns of the zeolites: (a) ZSM-12; and (b) MCM-22. (**B**) X-ray diffraction patterns of the Beta type zeolite. The reflexes due to the γ-Al_2_O_3_ phase are marked by asterisks.

The X-ray diffraction pattern of the ZSM-12 sample is shown in Figure 1A. The well-developed reflections characteristic of this structure (MTW) can be discerned. No other phases are present.

**Figure 2:**
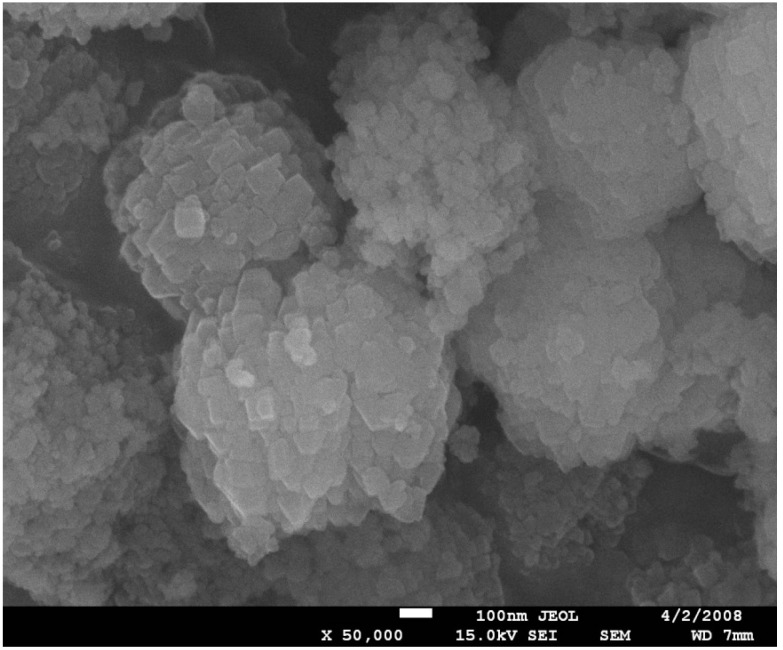
SEM microphotograph of ZSM-12 type zeolite.

A SEM microphotograph (Figure 2) reveals the presence of the *ca.* 1 µm spheroidal aggregates, consisting of smaller square, very thin plates *ca.* 80–100 nm. Some plates are not well-developed, with rounded edges, which might point to some amorphization of the zeolite.

**Figure 3:**
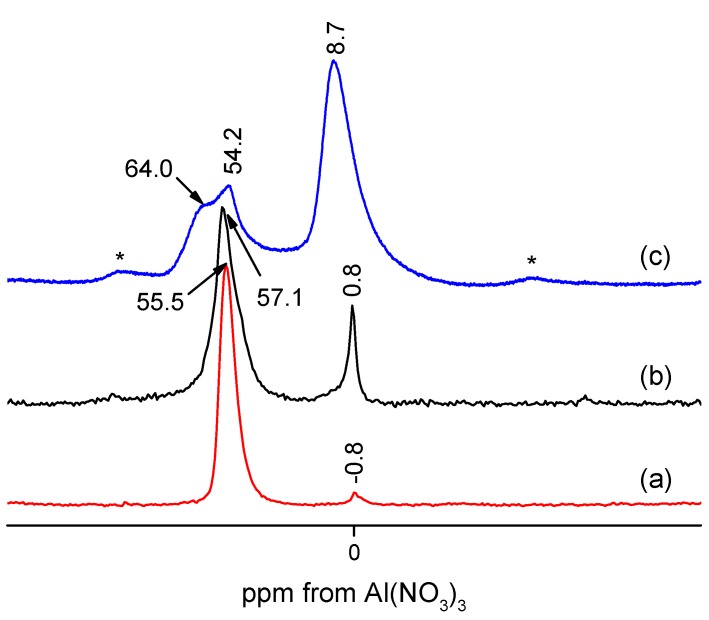
^27^Al MAS NMR spectra of the zeolite samples: (a) ZSM-12; (b) MCM-22; and (c) Beta. Spectra (a) and (b) were recorded at 300 MHz and spectrum (c) at 500 MHz. Asterisks denote spinning sidebands.

A representative ^27^Al MAS NMR spectrum of the zeolite is visualized in Figure 3a. As is seen, a very strong signal at 56 ppm points to the presence of tetrahedrally coordinated framework aluminium in the sample [14]. There is also a weak line at *ca.* 0.6 ppm due to the residual extra-framework aluminium with octahedral coordination. Most of aluminium, as shown in Figure 3a, is located in the zeolite tetrahedral framework positions, giving rise to bridging OH acid centres.

**Figure 4:**
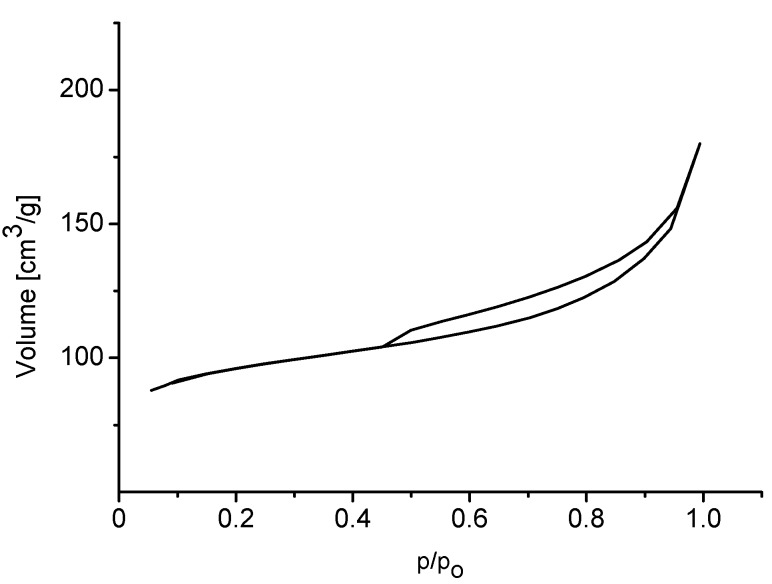
Adsorption/desorption isotherms of nitrogen on the ZSM-12 sample.

Studies of nitrogen sorption gave a typical adsorption-desorption isotherm depicted in Figure 4. The isotherm is of type IV and denotes the presence of mesopores. Finally, the shape of hysteresis loop points to the presence of inkstand-like pores in the sample. Details on adsorption of nitrogen are summarized in Table 1.

**Table 1:** Sorption properties of the ZSM-5, ZSM-12, MCM-22 and Beta type zeolites.

Sample	SSA ^a^ (m^2^/g)	S_E_^b^ (m^2^/g)	S_μ_^c^ (m^2^/g)	V_T_^d^ (cm^3^/g)	V_μ_^e^ (cm^3^/g)	V_m_^f^ (cm^3^/g)
ZSM-5	347	67	280	0.220	0.12	0.100
ZSM-12	342	103	239	0.279	0.10	0.642
MCM-22	418	93	337	0.518	0.16	0.691
Beta	442	304	138	1.081	0.08	1.010

^a^: multipoint BET specific surface area; ^b^: external surface (*t*-plot); ^c^: internal surface area (*t*-plot); ^d^: total pores volume; ^e^: micropores volume (*t*-plot); ^f^: mesopores volume. The t-plot method details given in [15].

#### 2.1.2. MCM-22

The framework density of silicon and aluminium atoms is 16.5 T atoms/1000 Å^3^ and there are 72 T-atoms in the unit cell. In the structure of MCM-22 zeolite there are large cylindrical cavities (7.1 × 7.1 × 18.2 Å) formed by 12-membered rings interconnected by the straight 10-MR (4.0 × 5.5 Å) channels. The interconnected sinusoidal channels (4.1 × 5.1 Å) form another bidimensional pore system. Moreover, in MCM-22 large 12-MR pockets are present with the dimensions of 7.1 × 7.1 × 9.0 Å. The pockets are located at the external surface of zeolite crystals.

**Figure 5:**
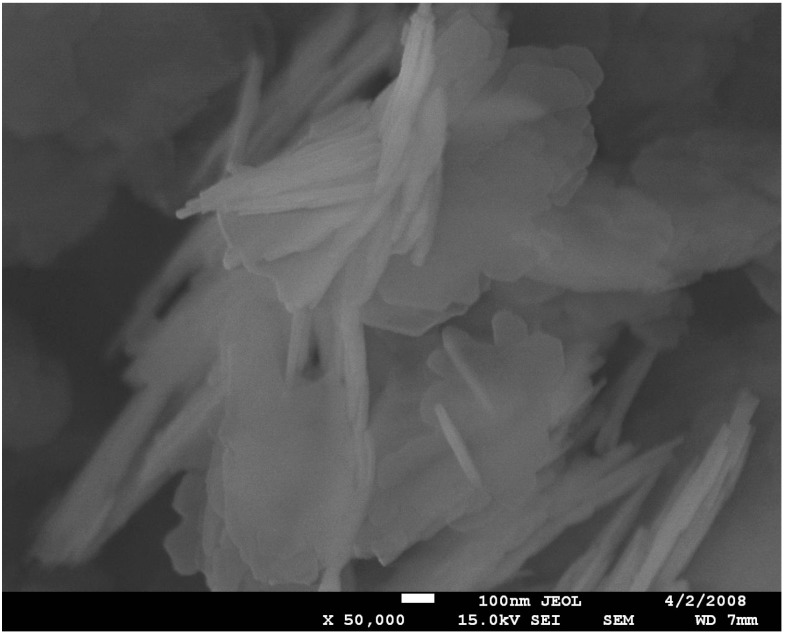
SEM microphotograph of MCM-22 type zeolite.

The MCM-22 material gives an XRD pattern with lines corresponding to the MWW structure (Figure 1B). Close inspection of the diffractogram did not reveal the presence of other phases. The sample exhibits a plate-like morphology, as it is seen in a SEM microphotograph (Figure 5). The dimensions of plates range from 20 to 50 nm.

**Figure 6:**
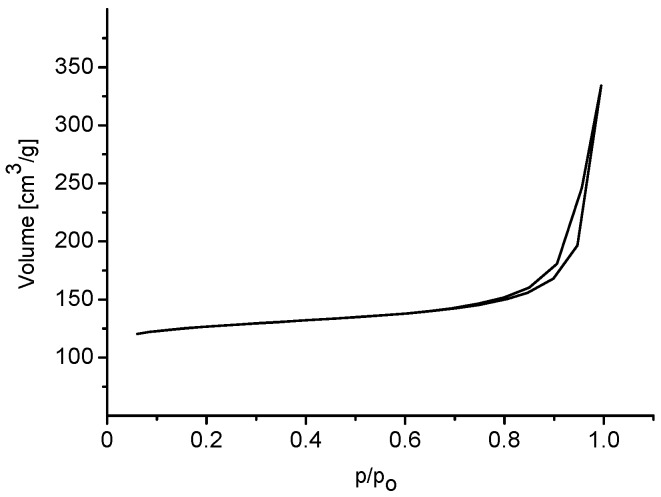
Adsorption/desorption isotherms of nitrogen on the MCM-22 sample.

The ^27^Al MAS NMR spectrum confirms the presence of tetrahedral, framework aluminium with a chemical shift of 56 ppm (Figure 3b). The relatively strong signal of extra-framework aluminium is also clearly seen at 0.7 ppm. The adsorption/desorption isotherm is of type IV, and the H3 type hysteresis loop reveals the presence of mesopores (Figure 6). The highest micropore volume of all zeolite catalysts studied was found for MCM-22 (Table 1). 

#### 2.1.3. Zeolite Beta

The framework density of silicon and aluminium atoms is 15.3 T atoms/1000 Å^3^ and there are 64 T-atoms in the unit cell. Zeolite Beta is a hybrid of two distinct structures. Both polymorphs possess three-dimensional 12-membered ring pore systems. The channel running along the 001 direction is smaller (5.5 × 5.5 Å), and the channel along the 100 direction is larger (7.6 × 6.4 Å). The zeolite Beta exhibits a high density of stacking faults and high concentrations of framework hydroxyl groups [16]. X-ray diffraction pattern of zeolite Beta is shown in Figure 1B. Zeolite Beta gives the most intensive reflexes at 7.87° and 22.50° 2θ, which are characteristic for the BEA type structure. There are also few other diffused signals which were assigned to the γ-Al_2_O_3_ phase (in Figure 1B the main reflections due to γ-Al_2_O_3_ were marked with asterisks.) Zeolite Beta was provided in the form of cylindrical extrudates (diameter Ø = 2 mm, length 8–10 mm)—aluminium oxide must have been used as an additional component during manufacturing of the zeolite extrudates. 

The ^27^Al MAS NMR spectrum of zeolite Beta is visualized in Figure 3c. Three signals were observed in the spectrum, at 64, 54.2 and 8.7 ppm, respectively. The tetrahedral framework aluminium due to zeolite Beta is seen at 54.2 ppm. The other signals were assigned to the γ-Al_2_O_3_ phase. Thus, the signal at 64 ppm, overlapped onto the 54.2 ppm one, is due to tetrahedrally coordinated aluminium, and octahedrally coordinated Al is seen at 8.7 ppm [17]. To conclude, ^27^Al MAS NMR spectroscopy confirms independently the presence of γ-Al_2_O_3_ phase in the sample.

Adsorption data for zeolite Beta reveal 442 m^2^/g specific surface area and high external surface area. The adsorption/desorption isotherm is of type IV and pronounced hysteresis loop reveals the presence of mesopores (Supplementary Material, Figure S3). The extrudates are characterized by a high mesopore volume, with the maxima located at 80 Å (majority) and 284 Å (DFT/Monte-Carlo pore volume distribution). Micropore volume is relatively low due to the presence of substantial amounts of the γ-Al_2_O_3_ phase (Table 1). 

### 2.2. Catalytic properties

Synthesis of perfluoroalkylglucosides by the Fischer reaction was carried out under conditions relevant for practical implementation, including an industrial scale manufacture of alkylpolyglucosides [7]. In addition to alkylglucosides, formation of oligomers is also observed due to further reaction with excess glucose molecules. The oligomerization level of the industrial products is usually *ca.* *n*_average_ = 1.2 – 1.5. Theoretically, one can expect formation of analogous oligomers when using perfluoroalcohols as starting materials. The two decisive factors are: (i) type of a catalyst used, and (ii) synthesis conditions. It is well known that lower temperatures (*ca.* 75 °C) facilitate the formation of glucofuranosides, while slightly higher ones (*ca.* 100 °C) lead preferentially to glucopyranosides. Consequently, syntheses of perfluoroalkylglucosides were carried out both at 70 and 100 °C under reduced pressure, *i.e.*, under the conditions known to yield higher conversion levels. Before the catalytic tests, zeolite materials were calcined to remove physically adsorbed water (*cf.* Experimental Section).

Generally, the conversion levels observed were around 10% (Table 2). Zeolite Beta, however, proved to be much more efficient catalyst, as 30% conversion of glucose was observed when using this catalyst. We conclude therefore that glucosidation by the Fischer reaction using perfluoroalcohols over acidic catalysts of different composition and properties proceeds with difficulty, generally giving low yields. This is due to the specific properties of perfluoroalcohols. Zeolite Beta has a well-developed mesopore system, with maxima located at 80 and 284 Å, which enables faster diffusion of reactants during the liquid-phase process. 

**Table 2:** Synthesis of perfluoroalkylglucosides over different heterogeneous catalysts at 100 °C. Reaction conditions: glucose: alcohol molar ratio = 1:10, *p* = 200 Torr, reaction time 5 h.

Catalyst	Conversion of Glucose (mol %)	Colour (Iodine Scale)
ZSM-5	11.1	<5
ZSM-12	10.0	<5
MCM-22	9.1	<5
Beta	30.0	60
P/CT122 ^a^ [15]	35.4	<5
Mont. KSF ^b^ [15]	30.0	<5

^a^: P–Purolite; ^b^: montmorillonite KSF.

**Figure 7:**
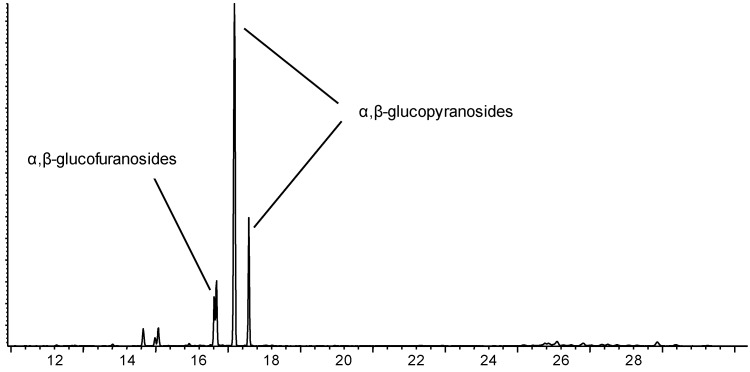
**A** representative GC chromatogram of octafluoropentylglucoside.

According to the mechanism of Fischer reaction, anomers of glucopyranoside and glucofuranoside will be present in the post-reaction mixture [18]. Usually four signals were detected, grouped in the two doublets, as shown in Figure 7. 

The assignments are shown in Table 3, and a comparison between the performance of zeolite Beta and other heterogeneous catalysts described elsewhere [19] is made. As it is seen, the selectivity towards glycosidation products is very similar over zeolite, clay mineral and ion-exchange resin, respectively. Over zeolite Beta, however, significantly higher amounts of α-glucopyranoside were observed (Table 3). 

One factor which might be importance for different classes of catalysts tested in the Fischer reaction is the acidity of the samples. Therefore, acidity was compared for the most active catalysts: the ion-exchange resin Purolite, the layered aluminosilicate montmorillonite and Beta type zeolite. Ammonia was used as a probe molecule, and Temperature Programmed Desorption (TPD) of ammonia was carried out under the same conditions and using similar portions of catalysts. In this way the TPD curves (Supplementary Material, Figure S1) which could be directly compared were obtained. Gaussian deconvolution of the NH_3_ signals allowed a convenient comparison of the acidity of the three samples studied, and the results are summarized in Table 4.

**Table 3:** Distribution of anomers in the glucosidation products obtained over three different type catalysts: zeolite Beta, the acidic cationite ion-exchange resin Purolite, and the KSF type montmorillonite.

Catalyst	α-GP ^a^	β-GP ^b^	α-GP + β-GP	α/β-GP ^c^	Σα, β-F ^d^
Zeolite Beta	60.56	15.48	76.04	5.22	18.74
P/CT122	56.87	24.09	80.96	2.36	19.05
Mont. KSF	63.78	17.98	81.76	3.54	18.24

^a^: α-glucopyranoside; ^b^: β-glucopyranoside; ^c^: α-glucofuranoside; ^d^: β-glucofuranoside.

**Table 4:** Relative population and distribution of acid centres in the most active catalysts, obtained from temperature-programmed desorption (TPD) of ammonia.

Sample	Relative Acidity of the Catalysts (a.u.)			
	Weak	Medium	Strong	Total
Purolite	3.0	32.9	-	35.9
Montmorillonite	1.1	2.3	-	3.4
Zeolite Beta	8.9	8.5	2.0	19.4

The lowest total acidity was found for montmorillonite, and the highest for Purolite. Medium-strength acid sites were observed predominantly in Purolite. Zeolite Beta, on the other hand, was characterized by a medium amount of acid sites, some of them were however strong (temp. of maximum ammonia desorption was 523 °C). There is no direct evidence of acidity influence onto the conversion level in the Fischer reaction (*cf.* Table 2). This is in accord with the earlier studies, where the amount of acid centres was not decisive for formation of butylglucosides over MCM-41 type materials [5]. Moreover, better activity was found for the catalysts containing medium strength acid sites [2]. Zeolite Beta is characterized predominantly by weak and medium strength acid sites (Table 4). Note, however, that the catalysts studied belong to very different classes of solids, and thus the composition of the pore system is different. This is manifested by various hydrophobic-hydrophilic properties of the surface available for reactants [2], and might be an interesting issue for further investigation. 

**Figure 8:**
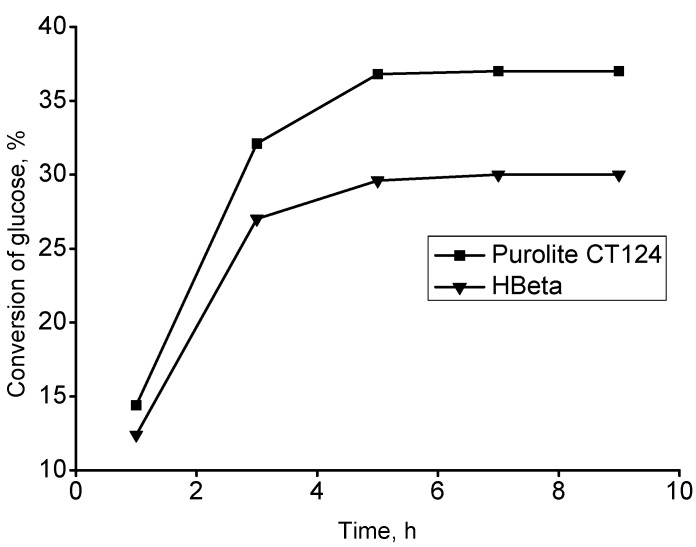
Conversion of glucose as a function of the synthesis time over Purolite CT124 and zeolite H-Beta at 100 °C.

Finally, it was also of interest to study the kinetics of the reaction over two different materials, Purolite CT122 and Beta zeolite, used as catalysts in glycosidation. Typical results are visualized in Figure 8. As it is seen, for both catalysts the conversion of glucose increases linearly until *ca.* 3 h, and then levels out giving a plateau after *ca.* 6 h of reaction. Formation of methylglucopyranoside after hydrolysis of levoglucosan tends to show similar characteristics, and the reaction is clearly approaching the equilibrium conversion [20]. 

## 3. Experimental Section

### 3.1. Preparation of the Samples

Synthesis of zeolite ZSM-12 was carried out using colloidal silica Ludox HS40, tetrabutylammonium bromide (both from Sigma Aldrich, Münich, Germany), sodium aluminate (Riedel de Haën, Seelze, Germany) and sodium hydroxide. Crystallization was performed under hydrothermal conditions in teflon-lined autoclaves at 160 °C for 6 days. The samples were washed with water and dried, giving material with SiO_2_/Al_2_O_3_ = 79 (XPS). MCM-22 was prepared using Cabosil M-5, sodium aluminate (Riedel de Haën), hexamethylenoimine (Sigma Aldrich) and NaOH. Synthesis was carried out 155 °C for 8 days (rotating autoclaves) and the resulting material had SiO_2_/Al_2_O_3_ = 49. Zeolites ZSM-5 and Beta were commercial products obtained from Zeolyst International (Conshohocken, PA, USA) and Tricat Zeolites GmbH (Bitterfeld, Germany), respectively. The two samples of ZSM-5 had SiO_2_/Al_2_O_3_ = 48 and 80, and the BET areas equal to 347 and 400 m^2^/g, respectively. The SiO_2_/Al_2_O_3_ = 21 molar ratio (XRF) and specific surface area of 442 m^2^/g were characteristic for the Beta type zeolite used (lot No. TZB-213). 

### 3.2. XRD Measurements

X-ray diffraction patterns were measured with Bragg-Brentano geometry using Cu Kα radiation, in the 2θ 2–50° range. SEM and TEM images as well as EDAX analysis were recorded on a JSM 7500F electron microscope (JEOL, Tokyo, Japan) working in TEM mode.

### 3.3. Sorption Properties

Adsorption of nitrogen adsorption at 77 K was studied using a Nova 2000 series analyzer (Quantachrome, Boynton Beach, FL, USA). Before the adsorption experiments the samples were heated in vacuum at 300 °C for 20 h. The multipoint BET method was applied to calculate the total surface area, while discrimination between micro- and meso-porosity was done by the *t*-plot method. The external surface area (S_E_), internal surface area (S_μ_), total pores volume (V_T_), and micropores volume (V_μ_) were determined by applying the *t*-method micropore analysis according to De Boer [14], and SiO_2_ was used as a reference material.

### 3.4. MAS NMR Measurements

Solid-state magic-angle-spinning (MAS) NMR spectra were acquired using the Apollo console (Tecmag, Houston, TX, USA) at the magnetic field of 7.05 T produced by the 300 MHz superconducting magnet (Magnex, Oxford, UK). A Bruker HP-WB MAS probe with the 4 mm zirconia rotor was used to obtain ^27^Al MAS NMR spectra at the spinning speed of 8 kHz. For ^27^Al spectra, a single 2 μs rf excitation pulse corresponded to π/6 flipping angle in the liquid, and 2000 scans were acquired with 1 s delay. The single-pulse ^27^Al MAS NMR spectrum of zeolite Beta was measured on an Avance 500 MHz spectrometer (Bruker, Karlsruhe, Germany) operating at a magnetic field of 11.7 T, using zirconia rotor spun at 12 kHz, 0.2 µs (π/15) short pulses with a repetition time of 0.3 s, and 6144 accumulations. The frequency scale in ppm was referenced to 1 M aluminium nitrate solution. 

### 3.5. Synthesis of Perfluoroalkylglucosides

Glucose (9 g, 0.05 mol), 1-octafluoropentanol (116 g, 0.5 mol) and a catalyst (3.6 g) were placed in a glass reactor. The mixture was stirred for 5 h at 100 °C under 200 Torr pressure. The unreacted glucose and the catalyst were separated by filtration, and the reaction products were obtained as a solution in fluoroalcohol. The alcohol was distilled off under reduced pressure on a rotary evaporator. Then 100 mL of water was added to the concentrated products and the remaining fluoroalcohol was removed as an azeotrope. In this way, the water solution of glucoside (containing 20% of non-volatile residue) was prepared. Next, the products were dried to give a pale-green viscous oil and analyzed by the GC/MS method. Analysis was performed using a HP 6890 series GC system (Hewlett-Packard, Palo Alto, CA, USA) equipped with a MSD detector (HP 5973 Network). A 30 m capillary column with 0.2 mm diameter, modified with HP-5M methylphenylsilicone, was used for separation of the reaction products. 

## 4. Conclusions

We have demonstrated that glycosidation using the Fischer reaction may be readily accomplished over a variety of zeolite catalysts containing acid sites. Thus, hydrogen forms of four different zeolites—ZSM-5, ZSM-12, MCM-22 and Beta—were used for this purpose. Among the zeolite samples studied, the best performance in terms of conversion (30%) was obtained over zeolite Beta. The activity of zeolite Beta was comparable with that of some non-zeolitic catalysts, the ion-exchange resin Purolite and the KSF type montmorillonite.

The Fischer reaction was studied using as substrates glucose and 1-octafluoropentanol. The corresponding perfluoroalkylglucosides were prepared for the first time. Such novel compounds might broaden the known class of fluorine-containing surfactants and could be of interest in future applications.

## Supplementary Materials

Supplementary Materials can be accessed at: http://www.mdpi.com/1420-3049/20/04/6140/s1.
