# Supplementary material for: Synthesis of Novel Perfluoroalkylglucosides on Zeolite and Non-Zeolite Catalysts
Source: Molecules. 2015 Apr 8;20(4):6140–52. doi: 10.3390/molecules20046140 (PMC6272255; doi:10.3390/molecules20046140)
Supplement: Supplementary file 1 [file molecules-20-06140-s001.pdf]

Supplementary Materials

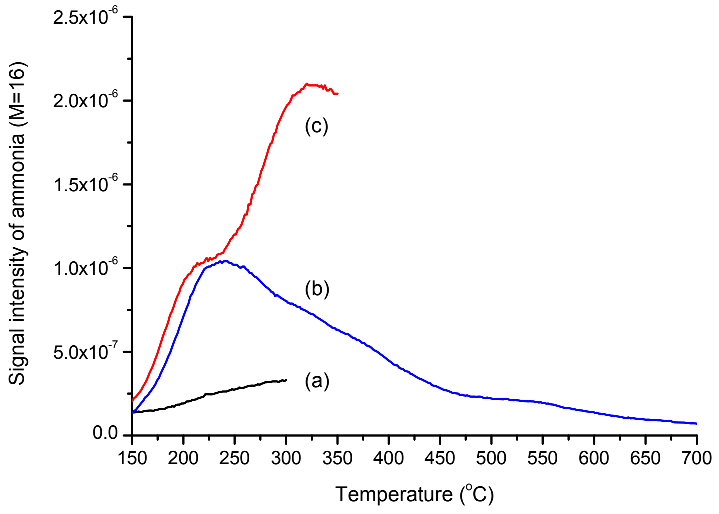

Figure S1. A comparison of thermodesorption curves of ammonia from: (a) montmorillonite; (b) zeolite Beta; and (c) ion-exchange resin Purolite.

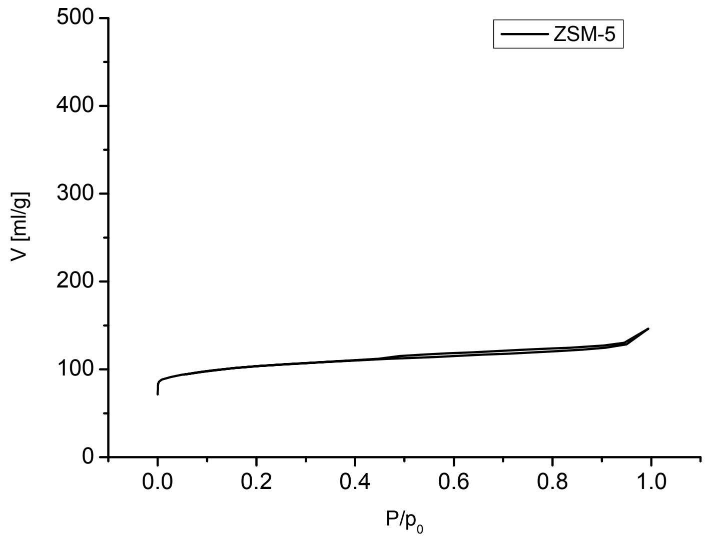

Figure S2. Adsorption/desorption isotherms of nitrogen on the ZSM-5 sample.

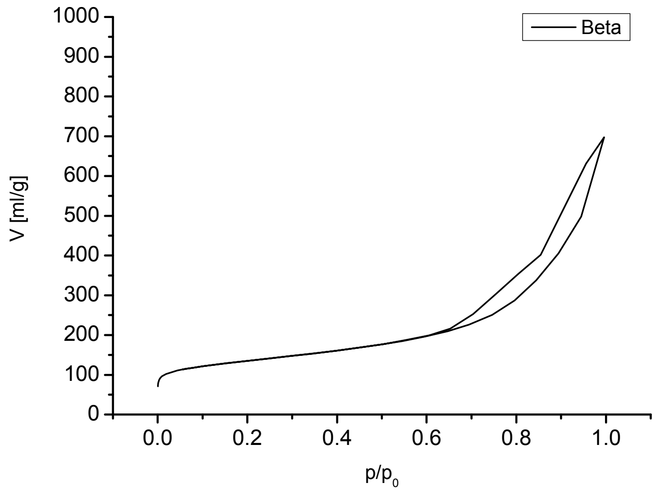

Figure S3. Adsorption/desorption isotherms of nitrogen on the Beta type zeolite.
